# Penile implant surgery-managing complications

**DOI:** 10.12703/r/10-73

**Published:** 2021-09-24

**Authors:** Axel Alberto Cayetano-Alcaraz, Musaab Yassin, Ankit Desai, Tharu Tharakan, Georgios Tsampoukas, Martina Zurli, Suks Minhas

**Affiliations:** 1 Andrology Department, Imperial College Healthcare NHS Trust, Charing Cross, London, UK; 2 Urology Department, Princess Alexandria Hospital, Harlow, UK

**Keywords:** Penile implantation, penile prosthesis, reoperation, erectile dysfunction surgery, preoperative care, risk factors, prosthesis implantation, instrumentation, glans ischemia, glans necrosis, inflatable penile prosthesis (IPP), prosthesis-related infections, salvage therapy, urethra

## Abstract

Penile prosthesis surgery represents the end-stage treatment for erectile dysfunction. It is conventionally used only in cases of erectile dysfunction refractory to pharmacological treatments or vacuum constriction devices. Contemporary literature suggests that penile prothesis surgery is associated with a high satisfaction rate and a low complication profile. However, it must be appreciated that the complications of surgery can have devastating consequences on a patient’s quality of life and satisfaction and include infection, prosthesis malfunction, penile corporal perforation and penile length loss. Several factors – such as appropriate patient selection, methodical preoperative assessment and patient optimization, specific intraoperative protocols and postoperative recommendations – can reduce the risk of surgical complications. This narrative review discusses the diagnosis and management of both intraoperative and postoperative complications of penile prosthesis surgery.

## Introduction

Penile prosthesis surgery represents the end-stage treatment for erectile dysfunction. It is conventionally used only in cases of erectile dysfunction refractory to pharmacological treatments or vacuum constriction devices. Contemporary literature suggests that penile prothesis surgery is associated with a high satisfaction rate and a low complication profile^1^. The complications of surgery can have economic ramifications (hospital admissions and revision surgery) and also negatively impact patient satisfaction and quality of life as there is a risk of penile length loss.

Although there is a paucity of high-quality data^2^, several factors – such as appropriate patient selection, methodical preoperative assessment, patient optimization, specific intraoperative protocols, and postoperative recommendations – may influence the risk of surgical complications. This narrative review discusses the prevention and management of both intraoperative and postoperative complications of penile prosthesis surgery.

## Prevention of complications

Table 1 summarizes preventive measures for complications in surgeries for penile prosthesis insertion. These measures are explained in detail in the following sections.

**Table 1.** Prevention of complications for penile prosthesis insertion.

	Comments
Surgeon’s experience	High-volume surgeons have fewer complications and revision surgeries.
Appropriate patient selection	Identification of patients more prone to complications (diabetes mellitus, history of spinal cord injury, polysubstance abuse, and HIV status).
Patient counselling	Comprehensive explanation of risks, establishing real expectations, and informing a tailored risk assessment.
Preoperative optimization	Smoking cessation, strict glucose control, and avoidance of skin infections.
Preoperative urine culture	Negative urine culture prior to the operation.
Hair removal	Clipper is preferred over razors.
Surgeon handwashing	No technique has shown better outcomes.
Prophylactic antibiotics	Ensure appropriate coverage of Gram-negative and positive organisms. Include antifungal prophylaxis in susceptible patients.
Infection control programs	Implementing safety checklists decreases infection rates.
Appropriate instruments	Decreases surgical times and allows effective problem-solving.
Prepping	Chlorhexidine scrubbing is recommended.
Glove change and double gloving	Switching gloves in critical steps of the operation could reduce infection rates. There is no direct evidence for double gloving.
Urethral catheterization	It prevents bladder injury during reservoir insertion. Early catheter removal is advisable.
No-touch technique	Minimizing skin contact decreases infection rates.
Intraoperative safety manoeuvres	Prevent and identify complications such as corporal perforation, urethral or bladder injury.
Antibiotic-impregnated or -coated implants	Lower the risk of postoperative penile prosthesis infection.
Postoperative drain placement	It prevents haematoma formation without increasing infection rates.

### Surgeon’s experience

Studies have reported that high-volume surgeons have shorter operative time and fewer iatrogenic failures (infection, erosion and poor positioning) and prosthesis removal procedures in comparison with low-volume surgeons^3,4^. Moreover, surgeons performing penile prosthesis more frequently have been shown to have higher revision-free survival time than less experienced surgeons^3,4^. Onyeji *et al*. reviewed data from the New York Statewide Planning and Research Cooperative System database and noted that 14,969 inflatable penile prosthesis (IPP) procedures were performed in the period of 1995 to 2014^5^. The authors observed that a volume of surgeries per year of more than 31 cases was associated with a decreased risk of requiring revisions for inflatable prosthesis infection (*P* <0.001)^5^. Furthermore, studies are needed to evaluate the required learning curve to perform both malleable and IPP surgery.

### Appropriate patient selection

In order to mitigate the risk of penile prosthesis infection, it is important to identify and try to prevent any risk factors.

***High-risk patients.*** In two studies in the last decade, the risk of revision surgery because of infection was higher in men with poorly controlled diabetes mellitus (DM) than in men without diabetes (1.88% vs. 1.53%; log-rank *P* = 0.0052)^6,7^. A recent meta-analysis reported that the presence of DM significantly increased the infection rate of penile prosthesis surgery (odds ratio [OR] 1.53, 95% confidence interval [CI] 1.15–2.04, *P* = 0.004)^8^. Therefore, improving glycaemic control through lifestyle changes and optimization of medical management may help reduce prosthesis infection rate.

Moreover, patients with a spinal cord injury were reported to have an increased risk of postoperative infections (9%)^9^. This has been postulated to be due to sensory loss causing poor handling of the prosthesis and delayed identification of complications such as impeding erosion^10,11^. Furthermore, constant urethral manipulation due to intermittent self-catheterization and a higher susceptibility to recurrent urinary tract infection have been reported to contribute to the higher risk of prosthesis infections in this patient cohort^10,11^.

Patients with a history of polysubstance abuse and homelessness had an increased risk of IPP infection in a single retrospective analysis^12^. The mechanisms that underly this association are unclear but were purported to be related to bacteriaemia due to drug use^12^. Therefore, it may be appropriate to ensure drug abstinence prior to prostheses surgery.

Penile prosthesis revision surgery is associated with a 10% increased risk of infection compared with primary insertions (0.1%)^13,14^, and patients should be counselled accordingly.

Given that many patients undergoing solid organ transplantation have comorbidities, including diabetes, hypertension and cardiovascular disease, there are concerns that this population will be at higher risk of prosthesis infection^15^. However, a recent systematic review of patients who had a solid organ transplant and penile prosthesis insertion did not show a greater infection risk (2.1% vs. 3.7%, *P* = 0.5) or non-infectious complications (9.8% vs. 4.7%, *P* = 0.08), although during revision surgeries there is a greater risk of injury (2.8% vs. 0%, *P* = 0.033)^15^.

A multivariate analysis of data from the American Premier Perspective database involving 5085 penile prosthesis procedures demonstrated that HIV (adjusted OR: 22.19, 95% CI 3.6–136.4, *P* <0.05) and diabetes (adjusted OR: 6.24, CI 1.23–31.77, *P* = 0.0276) were associated with infectious prosthesis explantations^16^. Thus, optimization of the aforementioned conditions could reduce the risk of infection.

Interestingly, there is mixed evidence for concomitant surgical procedures during IPP insertion and infection rates. For instance, when simultaneous artificial urinary sphincter implantation was performed, there was no increase in rates of explantation or revision^17^. For Peyronie’s disease, the infection rates were from 1 to 2% and revision rates were from 1 to 6% of the cases^18^.

***Patient counselling.*** There is evidence that appropriate patient counselling may improve postoperative satisfaction^19^. Kramer and Schweber observed a linear correlation between lower expectations and higher postoperative satisfaction in a cohort of 21 patients (*R* [Pearson] value was −0.489 and *R*^2^ was 0.239, *P* = 0.0245)^19^. Factors associated with improved patient satisfaction were psychological factors (positive emotions, self-esteem, confidence, enhancement of male identity, major live change, and self-image), improvement of sexual function, and relational factors (relationship improvement and the possibility of giving pleasure to the partner)^20^. Expectations such as penile length and sensation should be widely disclosed as they are associated with postoperative dissatisfaction^20^.

The authors emphasize the importance of informed consent in this setting. All patients should understand the operative risks, including infections and device malfunction. Patients should be counselled on the likely size and consistency of the erect penis and corresponding sensation and ejaculation changes. All patients should be aware of the potential postoperative pain, need for repeated operations, and alternative therapies^21^. Moreover, the patient should actively participate in selecting inflatable or malleable prostheses as it is essential to know the rationale behind each option, advantages and disadvantages, and cost differences.

Patients with Peyronie’s disease (relative risk [RR]: 4.2), a body mass index of greater than 30 (RR: 1.8)^7^, or previous radical prostatectomy (RR: 2.2) have a higher RR for lower satisfaction rates than the general implant population^7,22^. Therefore, these patient groups should be targeted for specific counselling to ensure that their expectations align with realistic prospects.

### Preoperative optimization of patients

***Smoking cessation.*** Cigarette smoking in surgical patients is associated with wound necrosis, delayed wound healing, surgical site infections (SSIs) and wound dehiscience^23^. The benefits of smoking cessation in reducing SSIs have been demonstrated in multiple randomized controlled trials^23^. Hence, smoking cessation should be advocated for all patients undergoing prosthesis surgery and this should be for a minimum of one month prior to the procedure to decrease the risk of infection^23^.

***Glucose control.*** As previously discussed, the presence of diabetes is associated with penile prosthesis infection and thus tight glycaemic control should be instigated prior to operative intervention. There is conflicting data on the relationship between haemoglobin A1c or serum glucose levels and infection rates^24–26^. Habous *et al*. suggested a preoperative HAbA1c target of below 8.5%; above this threshold, infection was predicted to occur with a sensitivity of 80% and a specificity of 65%^7^.

***Skin infections.*** Any skin infections or skin breaks in the groin or genital area increase the risk of infection of prosthesis infection and therefore antibiotic or fungal treatment should be started prior to any surgical procedure^27,28^.

### Preoperative treatments 

***Preoperative urine cultures.*** It is advisable to have a negative urine culture prior to any urological procedure where urine will be present. Because urethral catheterization is performed in penile prosthesis (IPP) insertion, there is a risk of bacterial contamination. A 2018 case series of 259 patients reported that the infection rate in IPP insertion or artificial urinary sphincter (or both) was 1% in patients without a positive urine culture^29^.

***Hair removal.*** An updated Cochrane meta-analysis regarding preoperative hair removal and rate of SSIs demonstrated that when it is necessary to remove hair, using a clipper is associated with fewer SSIs than razor use^30^. Moreover, the authors observed no difference in clipping the day before or on the day of the operation^30^. There is a lack of specific evidence pertaining to penile prosthesis surgery.

***Surgeon handwashing.*** An updated 2016 Cochrane systematic review showed that most evidence regarding the best antisepsis approach is of low or very low quality^31^. There is no convincing evidence that one technique is superior to others for reducing SSIs^31^. However, no specific studies for penile prosthesis insertion have been conducted.

***Prophylactic antibiotics.*** Several guidelines have advocated the use of prophylactic antibiotics prior to penile prosthesis surgery, and both the American Urology Association (AUA) and the European Association of Urology (EAU) committees have recommended that the antibiotic must have a treatment spectrum covering both Gram-negative and -positive organisms^1,32,33^. The International Consultation on Sexual Medicine has also specified that administration of antibiotics must occur at least one hour before incision^1^.

The AUA recommend an aminoglycoside plus a first- or second-generation cephalosporin or vancomycin for a duration of less than 24 hours^33^. In comparison, the EAU do not specify any specific regimens, owing to significant variability in antibiotic sensitivity and resistance patterns in Europe and worldwide, and have advised adherence to local microbiological guidelines^32^.

A multi-institutional study from 25 centres suggested that the current EAU and AUA antibiotic protocols may be inadequate because the micro-organisms identified in infected IPPs (n = 227) would be covered by prophylactic treatments in only 62 to 86% of cases^27^. Another multicentre study reported that the use of AUA prophylaxis guidelines was associated with more device infections (5.6% vs. 1.9%, *P* <0.01) and prosthesis removal (8.3% vs. 2.0%, *P* <0.001) compared with other non-conventional antibiotic regimes^34^. The authors also reported that the AUA-recommended antibiotic regimen was associated with a higher risk of device infection (OR 2.8, 95% CI 1.1–7.3) and explantation (OR 3.6, 95% CI 1.4–9.1). These findings suggest that institutions should adhere to local antibiotic sensitivity patterns and more research is necessary to identify an optimal prophylactic antibiotic regimen^34^.

Emerging data has highlighted that additional antifungal prophylaxis may be beneficial given that 12% of penile prosthesis infections are related to fungal infections, especially in patients with diabetes or obesity^35^.

There is no evidence to support the use of antibiotics postoperatively in the setting of penile prosthesis surgery and this practice risks antimicrobial resistance and antibiotic-related side effects^1,36^. Despite this, a study reported that about two-thirds of patients who had a penile prosthesis or artificial urinary sphincter were prescribed postoperative antibiotics^37^. Interestingly, in those prescribed antibiotics, the odds for device explantation were the same as the odds in those not given postoperative antibiotics (2.2% vs. 1.9%, *P* = 0.18)^37^. Some clinicians have advocated the use of postoperative antibiotics on the rationale that it may decrease the formation of biofilm on the penile prosthesis^38^.

## Intraoperatively

### Infection control programs

The risk of having postoperative infections is decreased through the use of strict internal protocols such as restricting the personnel entering or exiting the operating theatre, a specially trained team, appropriate handling of the equipment, and laminar flow ventilation. Also, the use of surgical checklists decreases the risk of postoperative infection^39^.

The authors recommend that all prosthesis procedure checklists should confirm the administration of intravenous antibiotics, aseptic methods (a minimum of five minutes of hand scrubbing for the surgeon, double-glove techniques, and 10 minutes of cleaning the patient with chlorhexidine followed by ChloraPrep), antibiotic irrigation of the prosthesis and minimal turnover of personnel^40^.

### Appropriate instruments

Having the appropriate set of instruments decreases the operative time and excessive manipulation of the tissue and prosthesis. Before the operation, checking the availability of the whole equipment is mandatory.

### Prepping

There is evidence that the use of chlorhexidine scrubbing is better than povidone-iodine products at reducing the SSI rate (9.5% vs. 16.1%, *P* = 0.004) and postoperative positive skin cultures (8% vs. 32%, *P* ≤0.05)^41,42^.

### Glove change and double gloving

Glove changing or double gloving has been recommended for penile prosthesis insertion. However, most of the evidence stems from orthopaedic research. For example, in prosthetic joint surgery, it has been found that switching gloves could reduce infection rates after draping, before manipulating prosthetic materials, in prolonged operations, and if glove perforation is identified^43^. There is no direct evidence for double gloving^43,44^.

### Urethral catheterization

No study has investigated whether urethral catheterization decreases perioperative complications. However, catheterization is frequently used in penile prosthesis surgery on the rationale that it will decompress the bladder before the reservoir insertion in inflatable prosthesis and can be a valuable tool in identifying the urethra and thereby preventing urethral injury during corporal dissection. A Cochrane systematic review investigating the use of short-term urinary catheterization following urogenital surgeries showed fewer urinary tract infections when a catheter was removed earlier (1 vs. 3 days, RR 0.50, 95% CI 0.29–0.87)^45^.

### No-touch technique and intraoperative safety manoeuvres

There is evidence that minimizing the contact between the prosthesis and the patient’s skin through the use of additional draping decreases infection rates (2% vs. 0.45%)^46^. This technique aims to ensure that the implant, instruments, and the surgeon’s hands are never in direct contact with the patient’s skin^1^.

There are well-known intraoperative safety manoeuvres that might decrease the chances of complications or allow early detection and prompt treatment. These surgical steps include the following:

•    Field goal test to rule out proximal corporal perforation

•    Interrogation sign test to rule out distal corporal perforation

•    Distal irrigation test of each corporotomy to rule out urethral perforation

•    Lateral dilatation of corpora

•    Deflation of the bladder before reservoir insertion.

### Antibiotic-impregnated/coated implant

The use of antibiotic-impregnated implants has been shown to lower the risk of postoperative infection. A meta-analysis including 14 clinical case studies reported that the infection rate was lower for coated compared with non-coated protheses (2.32% vs. 0.89% vs. 2.32%, *P* <0.01)^47^. In regard to antibiotic combinations, the infection rates were 4.42%, 1.11%, 0.63%, and 0.55% for vancomycin/gentamycin immersion, hydrophilic coating, minocycline/rifampin (InhibiZone™) and rifampin/gentamycin immersion respectively^47^.

### Postoperative drain placement 

Postoperative drain placement has been used following penile prosthesis surgery to prevent haematoma formation (2–5% of cases). There is evidence that the infection rate is similar with or without closed suction drainage^48^.

## Complications

The most common penile prosthesis complications and their estimated prevalence are shown in Table 2. The following section focuses on how to identify and treat each of these complications.

**Table 2.** The most common penile prosthesis complications and their estimated prevalence.

Complication	Estimated prevalence
Infection of prosthesis - Virgin implantation - After revision	1–4%^13^ 10%^13^
Crossover	−0.6%^52^
Corporal perforation	1.1%^52^
Urethral injury	0.7%^52^, 1.6%^53^
Mechanical failure	3.1–19%^14,54,55^
Corporal erosion	3%^56^, 5%^54^
Revision surgery	5%^54^
Glans supersonic transporter deformity	5%
Reservoir herniation	0.7%^57^
Scrotal haematoma	1.3%^52^

### Corporal crossover

The trauma of a dilator / Furlow / prosthetic cylinder into the contralateral corpora through the septum can perforate the septal wall and cause corporal crossover. This can occur at either proximal or distal locations^49^. It is believed that corporal crossover occurs more frequently with infrapubic or subcoronal incisions than with the penoscrotal approach^49^. The causes for corporal crossover include corporal fibrosis and technical errors^50^.

Corporal crossover can be mitigated intraoperatively by always dilating each corpora laterally toward the 2 and 10 o’clock positions^1^. Corporal crossover is identified with the goal field test, which is confirmatory in the presence of asymmetrical corporal length on inserting dilators into the proximal corpora^50^. There should be an identical length or a minimal length difference between corporal; if ‘clanking’ of the dilators is felt or heard during this manoeuvre, a high suspicion of crossover should be considered.

Other signs of corporal crossover are that the second cylinder is difficult to insert, the urethral catheter is not situated in the midline when the prosthesis is activated, or there is an atypical penile appearance during inflation^21,49,51^.

Managing this complication entails keeping a wide dilator on the corpora without crossover while attempting to dilate the contralateral corpora more laterally and measuring the length again to confirm appropriate placement^21,49,51^.

Corporal crossover can be identified postoperatively if there is a tilted glans or an asymmetrical erected penis when the cylinders are inflated. Moreover, corporal crossover is typically confirmed postoperatively with magnetic resonance imaging (MRI)^50^ and the treatment choice is surgical correction.

### Corporal perforation

There are two types of corporal perforation: distal and proximal. Distal perforation to the urethra is suspected when the dilator, irrigation fluid or blood is seen protruding through the urethral meatus. The conventional management of this complication is to abort the procedure and reattempt surgery in a minimum of six months^21^. In some cases, only the contralateral cylinder is inserted, and revision surgery is performed in a delayed fashion^1,21^. This complication can be identified through the distal fluid challenge test, which involves flushing the corporotomy on each side with irrigation fluid^1^. The authors argue that the distal fluid challenge test should be incorporated in all safety checklists to allow early detection of this complication.

Proximal perforation is observed when there is a sudden loss of proximal corporal resistance during dilatation or when a very low position of the ipsilateral cylinder is noted^21^. Typically, correcting the path involves using scissors or direct visualization when dilating laterally towards the pelvic bone^1^. The management of this complication requires fixing the rear tip to the lateral edge of the corporotomy, so the cylinder will remain in place after closure, allowing a second intention healing of the disrupted corpora. An alternative approach would be to use a rear-tip sling, consisting of anchoring the cylinder’s proximal end with a non-absorbable suture to each side of the corporotomy^1,21^.

### Urethral injury

Urethral injury usually occurs during cavernosal dilatation and can be in the proximal or distal locations. Risk factors for urethral injury include revision surgery and fibrotic corpora (e.g., infections, diabetes, Peyronie’s disease or priapism)^58^. Urethral injury has also been described after penile straightening manoeuvres when correcting penile curvature^58^.

The main preventative steps for urethral injury include appropriate surgical exposure, urethral catheterization for easy anatomical identification, and lateral dilatation of corpora. Moreover, appropriate instrumentation is fundamental to facilitate corporal dilatation^58,59^. In cases of penile modelling (such as Peyronie’s disease), the risk of urethra injury can be decreased by placing the bending hand on the shaft while the other hand is compressing over the corporotomies^58^.

The recognition of urethral perforation can be difficult, so it is recommended that clinicians take extra caution to look for potential signs such as urethral bleeding, a visible dilator on the urethral meatus or prosthesis cylinder, or leakage of irrigation solution out of the urethra after instilling the corpora through the corporotomy^58,59^. If there are any concerns regarding urethral injury, confirmation can be made through cystoscopy^60^.

The intraoperative management of urethral injury includes repairing the defect and either deferring or completing the procedure; this decision depends on multiple perioperative factors. If there is a proximal perforation, immediate urethral repair accompanied by primary implantation and urinary diversion with a suprapubic catheter is suggested^58^, and abandoning the procedure should be considered if the injury is closer to the urethral meatus^60^. Perito described a staged technique for repairing distal urethral perforations entailing intentional hypospadias of the meatus and later secondary closure^61^.

However, when a urethral injury occurs and one or both corpora are dilated, abandoning the procedure may end in irreversible corporal fibrosis and penile shortening^59^. Consequently, some clinicians have advocated for the insertion of a temporary malleable prosthesis after salvage washout and later a definitive inflatable prosthesis^58^.

### Cylinder erosion / extrusion

Cylinder erosion is an externalization of the cylinders caused by a gradual weakening of the tunica albuginea^1^. This tunical deterioration could be instigated by excessive dilation of the corpora or a long-standing pressure due to oversized cylinders. Patients at a higher risk of this complication are those with diminished penile sensitivity (e.g., diabetics, paraplegics and radiation), previous prosthesis replacement, long-standing prosthesis and frequent urethral instrumentation^21,56,62^.

Cylinder erosion typically presents with the prosthesis protruding through the glans, urethral meatus, or distal penile shaft. Urethral erosion is characterized by dysuria, urethral discharge, early prosthetic infection and glans^58^.

The treatment of cylinder erosion is dependent on the clinical context, surgeon preference and experience. The most convenient option is to remove the cylinders and, after 4 to 6 weeks, replace the prosthesis^1^. Others perform a salvage washout, remove the prosthesis and immediately insert a malleable prosthesis on the non-eroded corpora.

Impending erosion, or extrusion, is when the cylinder is underneath the penile skin without penetrating it and can be treated using distal corporoplasty, involving the reposition of the cylinder tip into a new corporal cavity^21^.

### Glans necrosis

Glans necrosis is caused by a disruption of the glans blood circulation (dorsal arteries and terminal branches of the spongiosal arteries). Although the incidence of glans necrosis has not been reported, anecdotal evidence suggests that it is a rare complication of penile prosthesis surgery. The cavernosal arteries are injured during the prosthesis insertion, restricting the arterial supply to the dorsal and bulbourethral arteries^63,64^.

The risk factors for glans necrosis include dismantling procedures for penile lengthening, severe atherosclerotic cardiovascular disease, DM, smoking, past prosthesis removal and pelvic radiotherapy^63,65^. The perioperative factors associated with glans necrosis are a circumcoronal incision, aggressive penile degloving, and skin-tight penile dressings^63^.

Glans necrosis presents with a necrotic or discoloured appearance of the glans, absent or diminished glans capillary refill, and blisters^64^. The immediate removal of the prosthesis may preserve the glans, and conservative management usually leads to glans loss^63,64^.

### Bladder injury

Bladder injury is a rare complication of penile prosthesis surgery, and risk factors include blind reservoir insertion, penile prosthesis revision surgeries, previous pelvic surgery, and pelvic radiotherapy^66^. Bladder injury can also occur as a result of a gradual erosion into the bladder^67–70^. The risk of perforation is decreased by routinely draining the bladder before reservoir insertion, direct inspection during reservoir placement or ectopic submuscular reservoir insertion^1,71^. The clinical presentation of a bladder injury is haematuria after the procedure and can be confirmed with a computed tomography (CT) scan, retrograde cystogram or flexible cystoscopy^67,70^. The treatment of a bladder injury is a surgical exploration through an abdominal approach with repositioning of the reservoir and repairing the bladder^70^.

### Visceral injury

Visceral injury can occur during the reservoir insertion^72,73^. The risk factors are any previous pelvic surgery or local radiotherapy. The management of a visceral injury includes aborting the penile implantation procedure and immediate treatment of the sustained injury (e.g., bowel repair)^71^. It is worth noting that no bowel injury has been reported with the ectopic reservoir technique^74^.

### Vascular injury

Major vascular injuries are a rare complication and usually occur when the reservoir pouch is being developed. Given the proximity of large vascular structures to the external inguinal ring, performing a careful dissection is mandatory. The external iliac vein distance from the inguinal ring is 2.5 to 4 cm at a 20- to 60-degree lateral measurement from the inguinal ring^75^. Therefore, avoiding deep lateral dissection to the inguinal ring is important^75^. The management of a vascular injury should initially be direct compression, adequate exposure, and repair by a trained specialist with the requisite expertise^51^.

### Haematoma

The scrotum is the most common location for haematoma formation and this is due to an absence of compressive forces to abate any local bleeding. Haematomas can present in the immediate postoperative period or in a delayed fashion^76^. The management of haematomas is usually conservative, and scrotal exploration is seldom required^68,76^. Conservative management consists of bed rest, scrotal elevation, compressive dressing, application of ice, and antibiotics^68,76^. In order to mitigate the risk of haematoma formation, there should be an emphasis on meticulous haemostasis, compressive dressing, partial activation of the inflatable prosthesis, and a closed drain insertion^76,77^. Before and after the operation, paying attention to when to hold and start anticoagulation or antiplatelet drugs is essential^76^.

### Infection

The predominant organisms causing infection consist of skin flora and enteric organisms^27^. The clinical signs of infection include persistent scrotal or penile pain, signs of local (e.g., erythema and oedema) or systemic (e.g., fever and rigors) infection, pump attached to the scrotal skin, and purulent or serous discharge from the wound^1,78^.

Suspected infections should be treated with long-term oral antibiotics (10 to 12 weeks)^1^ and conservative treatment in a specific cohort of patients (no signs of sepsis, fever or leucocytosis), and localized prosthesis infection has been reported to be successful in 89% of cases^79^. However, if the pain persists or symptoms return despite antibiotic treatment, surgical exploration is advocated^1^. If the device infection is confirmed, hospital admission is recommended to administer broad-spectrum intravenous antibiotics, and appropriate cultures should be taken^1^. Moreover, removal of the entire prosthesis, followed by a washout of the wound with antiseptic solutions, is recommended. Following resolution of the infection, implantation of a new prosthesis can be performed following a delayed period of 3 to 6 months.

Mulcahy described a protocol for a salvage procedure, which has been modified to include the following recommendations: complete removal of all components, vigorous sequential washout using different antiseptics, a new implant insertion (usually a malleable prosthesis) and a course of antibiotics for 2 to 4 weeks^80^. The success rate of this salvage procedure technique has been reported to be 84 to 93%^80,81^. However, the contraindications for a salvage procedure are ketoacidosis, severe sepsis, abundant necrotic tissue, rapidly evolving infection, or an exteriorized implant^1,71^. It is worth noting that the mean penile length loss after delayed reimplantation is 3.7 cm (CI 95% 2.9–4.5) compared with a loss of 0.6 cm (95% CI 0.20–1.1) if a salvage procedure is successful^82^.

### Mechanical failure

The reported penile prosthesis survival rates at 5 and 10 years are 90.8% and 85% respectively^14^. Despite technological advancements in the development of implants, there is still a risk of mechanical failure. The malfunction of a prosthesis often requires replacement of the entire device^83,84^. Malleable implants carry the lowest mechanical failure complication rate because of their simplicity. Common mechanical failures are fracture of the tubes, a dysfunctional cylinder or reservoir, aneurysm of the cylinder and disruption of the connector. Both CT or MRI may be able to visualize the fracture, and the latter can also provide information on the integrity of the tunica^85^. If the complication has arisen early after the insertion, replacement of the dysfunctional cylinder is recommended. However, in late presentations, replacement of the whole device is the treatment of choice^86^.

The majority of mechanical failures are caused by fluid loss from the different penile prosthesis components^14^. The reservoir, any dysfunctional unit or the whole device needs replacement following the necessary precautions to avoid infection.

There is evidence that the reservoir placement in alternative sides is not associated with an increased mechanical failure rate compared with the retropubic space of Retzius^87^.

Aneurysm of the cylinder (as evidenced by bulging of the inflatable cylinder) is less common with advanced manufacturing but can still occur^88^. The appearance of a palpable lump after the deflation of the cylinder is characteristic of this complication. In cases when the aneurysm is large and bothersome, revision surgery has been reported to obtain excellent results^89^.

### Floppy glans

Glans hypermobility or Concorde deformity represents an infrequent malpositioning complication of penile prosthesis implantation. Although the incidence of this complication is low, the condition can cause severe patient dissatisfaction owing to painful intercourse, difficulties in penetration, and poor cosmesis^83,84,90^. Incorrect intraoperative prosthetic cylinder positioning or sizing can result in inadequate compression of the venous system between the Buck fascia and the corpora cavernosa, even when the cylinders are maximally inflated. As the surrounding tissues cannot efficiently restrict the blood flow, glanular tumescence becomes difficult to achieve. This phenomenon might occur particularly in patients with severe erectile dysfunction who have poor glanular blood flow at baseline or uncircumcised men for unknown reasons^91,92^.

There is evidence that the presentation of floppy glans syndrome was significantly higher in men who underwent implantation with the infrapubic approach than the penoscrotal approach (9.61% vs. 1.35%)^84^.

The diagnosis of a floppy glans is through clinical assessment and further imaging is considered unnecessary^85^. Although reconstructive glans fixation (glanulopexy) is considered the standard approach, some authors report that the formation of a capsule around the tip of the cylinder often resolves this deformity^92–94^. Glanulopexy involves suturing the glans against the cylinder heads and tying it to the tunica albuginea of the corpus cavernosum adjacent to the cylinder tip in one or more areas^86,95^. If the length of the cylinders is unacceptably short, placement of rear-tip extenders most likely will reverse the deformity. On the other hand, in the proximal migration of a unitary implant, the device has to be replaced, and the rear-tip extender needs to be stabilized by a sling suture or non-absorbable suture firmly attached to the tunica albuginea^92^.

### Reduced penile length / sensation

Decreased penile length is reported as one of the most common complaints of penile prothesis surgery. Up to half of the patients might report subjective loss of penile length irrespective of the technique^96^. This highlights the importance of accurately measuring penile length prior to any operation^97,98^.

An in-depth study of the anatomy and consideration of additional pathology such as Peyronie’s disease should guide all procedures. The use of ventral phalloplasty, suprapubic lipectomy, and liposuction and suspensory ligament release are standard techniques for increasing perceived penile length size^99^. These procedures can be performed simultaneously with the implant insertion, thereby reducing operating times and additional costs.

Other techniques such as relaxing tunica albuginea incisions, lengthening procedures such as the sliding technique or the use of postoperative penile rehabilitation with a vacuum device can also increase penile length and improve patient satisfaction^100^. Special consideration must be given in men with Peyronie’s disease undergoing penile implant insertion as defective penile length is a common complaint in this group of patients. The penile remodelling is indicated when a 30-degree residual curvature is noted after cylinder placement^101^. Additional grafting might also be necessary if remodelling does not achieve the expected results^65,102^. Further advanced application or sliding techniques may be necessary to restore the penile length. The authors advise that complex procedures should be performed in specialized centres with high-volume surgeons^103^.

## Conclusions

An understanding of the potential complications of penile prosthesis surgery is fundamental to optimize surgical outcomes and patient satisfaction. Taking appropriate preventive measures and knowing how to deal appropriately with any penile implant complication are paramount.
